# Predicting Rice Lodging Risk from the Distribution of Available Nitrogen in Soil Using UAS Images in a Paddy Field

**DOI:** 10.3390/s23146466

**Published:** 2023-07-17

**Authors:** Nozomi Kaneko Sato, Takeshi Tsuji, Yoshihiro Iijima, Nobuhito Sekiya, Kunio Watanabe

**Affiliations:** 1 Graduate School of Bioresources, Mie University, Tsu 5148507, Japan; 2 Office SoilCares, Yokkaichi 5100035, Japan; 3 Tsuji Farm Co., Ltd., Tsu 5140126, Japan; 4 Graduate School of Urban Environmental Sciences, Tokyo Metropolitan University, Hachioji 1920397, Japan

**Keywords:** spatial statistics, raster calculation, soil fertility map, multispectral image, smart agriculture

## Abstract

Rice lodging causes a loss of yield and leads to lower-quality rice. In Japan, Koshihikari is the most popular rice variety, and it has been widely cultivated for many years despite its susceptibility to lodging. Reducing basal fertilizer is recommended when the available nitrogen in soil (SAN) exceeds the optimum level (80–200 mg N kg^−1^). However, many commercial farmers prefer to simultaneously apply one-shot basal fertilizer at transplant time. This study investigated the relationship between the rice lodging and SAN content by assessing their spatial distributions from unmanned aircraft system (UAS) images in a Koshihikari paddy field where one-shot basal fertilizer was applied. We analyzed the severity of lodging using the canopy height model and spatially clarified a heavily lodged area and a non-lodged area. For the SAN assessment, we selected green and red band pixel digital numbers from multispectral images and developed a SAN estimating equation by regression analysis. The estimated SAN values were rasterized and compiled into a 1 m mesh to create a soil fertility map. The heavily lodged area roughly coincided with the higher SAN area. A negative correlation was observed between the rice inclination angle and the estimated SAN, and rice lodging occurred even within the optimum SAN level. These results show that the amount of one-shot basal fertilizer applied to Koshihikari should be reduced when absorbable nitrogen (SAN + fertilizer nitrogen) exceeds 200 mg N kg^−1^.

## 1. Introduction

Lodging has decreased the yield and quality of rice. In Japan, Koshihikari is the most popular rice variety and a favorite of the Japanese for its short, sticky grain and good taste. Although Koshihikari has long been cultivated in a wide area of Japan since it was developed in 1956, it is prone to lodging because of its long stems and high-yield features [1].

Rice lodging causes serious negative effects. Lodged rice plants shade themselves, preventing light from reaching the lower part of the plant. This inactivates photosynthesis, resulting in further yield loss and lower quality [2]. Additionally, they increase the workload of a combine harvester [3].

Excess nitrogen is a factor associated with rice lodging. High soil nitrogen concentrations weaken culm tissues, making them more susceptible to lodging [4]. Higher nitrogen fertilizer applications weaken the bending stress of japonica rice by reducing the thickness of the secondary cell walls in culm tissue [5]. The local cultivation guidelines for Koshihikari prioritize nitrogen management and recommend four to six meticulous top dressings. This allows Koshihikari to grow, avoid lodging, and maintain quality and yield [6,7,8].

There are two major reasons why excess nitrogen may be supplied. The first is the unverified distribution of available nitrogen in soil (SAN) in a paddy field. The Ministry of Agriculture, Forestry and Fisheries (MAFF) defined the optimum SAN concentration as 80–200 mg N kg^−1^ and recommended reducing the basal fertilizer when SAN exceeds the optimum level [9]. However, the spatial distribution of SAN is unknown, making it difficult to apply the appropriate amount of nitrogen fertilizer. Moreover, many Japanese commercial farmers prefer to apply one-shot basal fertilizer at transplant, which does not adjust the amount of fertilizer according to rice growth. The second is overlapping applications of fertilizer by the transplanter. When farmers apply a one-shot basal fertilizer, they may overlap application at the headland and on the edge of the paddy field.

Controlling the amount of one-shot basal fertilizer based on SAN is important to avoid lodging. Nevertheless, there is a lack of knowledge about the relationship between rice lodging and SAN. This hinders guidance on the appropriate use of one-shot basal fertilizer.

The current survey methods for rice lodging are visual observation and interviews with farmers to assess severity and distribution [10]. Remote sensing technology using unmanned aircraft systems (UASs) has recently been developed in agricultural research. It has advantages in cost effectiveness, area coverage, high-resolution imagery and flexibility in timing of data acquisition [11,12,13]. Combined with machine learning, it has been widely studied in precision agriculture, such as crop monitoring, crop classification, yield prediction and plant disease detection [14,15,16,17,18]. Attempts are being made to use UAS remote sensing to verify crop lodging. Chauhan et al. [19] reported that satellite/airborne-based remote sensing technologies using visible and near-infrared or microwaves have been researched to detect lodging and assess damage to crops. Tanaka and Kondoh [20] created a rice lodging risk map using a digital surface model (DSM), which was acquired during the panicle formation stage and 14 days before tillering. Yang et al. [21,22,23] approached rice lodging assessment using a DSM and texture information, and segmented rice lodging areas by deep learning of RGB images and vegetation indices in large fields.

Remote sensing research on physicochemical soil properties has been conducted by analyzing satellite images over a wide area for digital soil mapping. Mulder et al. [24] reported that satellite remote sensing is widely applied to the soil to determine texture, moisture, iron, organic carbon, salinity and carbonate contents by mixed kriging, classification and regression tree analytical methods. Machine learning techniques have been recently introduced to soil remote sensing. Zhang et al. [25] and Zhou et al. [26,27] applied machine learning methods to assess soil fertility, such as soil organic carbon and soil total nitrogen. In a UAS-based method focusing on a small area for precise analysis, Morishita and Ishitsuka [28] attempted to construct multiple soil physiochemical properties using random forest regression in a soybean field. Niwa et al. [29] estimated SAN by regression analysis in a wheat field, and Togami et al. [30] developed a SAN estimating formula for paddy fields using spectral reflectance and the measured total carbon (TC) content by multiple regression analysis. However, no study has estimated the SAN distribution in a paddy field using UAS and assessed the severity of rice lodging using the estimated SAN value.

The objective of this study was to predict the risk of rice lodging in a Koshihikari paddy field applied with one-shot basal fertilizer using UAS images. The spatial distribution of rice lodging severity and SAN content were evaluated, and their relationship was clarified. Additionally, a fertilizer control strategy was proposed based on the SAN distribution and transplanting operation.

## 2. Methods

### 2.1. Field Survey

#### 2.1.1. Study Field

**Figure 1:**
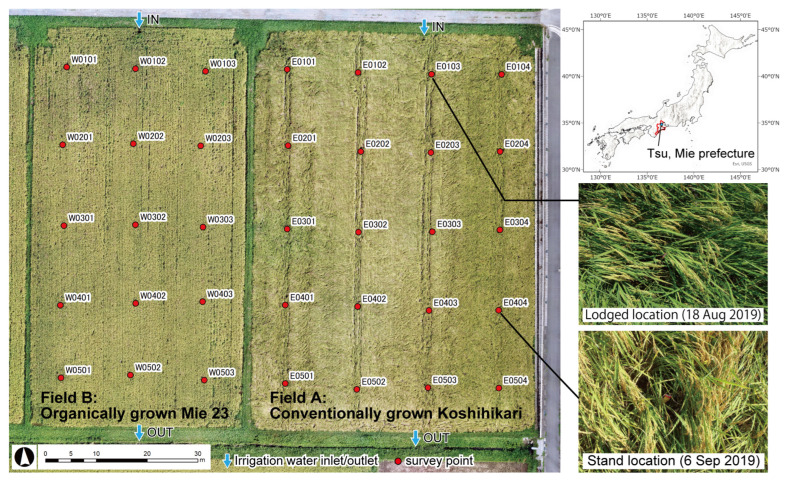
Location of the study fields and survey points.

We surveyed two adjacent paddy fields at Tsuji Farm (34°46′ N, 136°29′ E) in Tsu, Mie Prefecture, Japan, as shown in Figure 1. The mean annual temperature for the past 10 years in this area was 16.5 °C, and the mean annual precipitation was 1687 mm [31]. The main study field was Field A, with an area of 55 × 80 m, where Koshihikari was conventionally grown. Field B, with an area of 40 × 80 m, where Mie 23 was organically grown, was an additional study field to develop the SAN estimating equation. Irrigation water inlets and outlets were located at the northern and southern ends, respectively, as indicated by the blue arrows in Figure 1. The research period was from March to September 2019. The mean annual temperature and annual precipitation in 2019 were 16.9 °C and 1630 mm, which were the same level as in a normal year. In Field A, 2400 kg ha^−1^ of rice bran was applied as organic material in the winter, and 250 kg ha^−1^ (70 kg N ha^−1^) of one-shot basal chemical fertilizer “M cote 2877” was applied in May 2019. M cote 2877 was loaded on a transplanter and applied evenly at the same time as the Koshihikari was planted. In Field B, 1000 kg ha^−1^ of rice bran was applied similarly to Field A, and 900 kg ha^−1^ (63 kg N ha^−1^) of soybean waste was applied as the basal fertilizer in May 2019. Beginning in mid-August, Koshihikari started to lodge on the north side of Field A.

#### 2.1.2. Rice Growth Survey

Plant length, plant height and the SPAD value were measured on three plants at the 35 locations shown in Figure 1. The SPAD value was measured with the SPAD-502Plus digital chlorophyll meter (Konica Minolta, Tokyo, Japan). The normalized difference vegetation index (NDVI) was measured using the GreenSeeker Handheld Crop Sensor (Trimble Inc., Sunnyvale, CA, USA) at the same locations. NDVI is commonly used to diagnose plant health in remote sensing studies because it is a non-destructive and rapid measurement method [32]. Especially in rice research, it has been used in breeding, nitrogen use efficiency monitoring, yield prediction and growth stage identification [33,34]. Nine plants were taken at each location on 6 September 2019, for a yield survey. The Real Time Kinematic—Global Navigation Satellite System (RTK-GNSS) receiver, REACH (Emlid, Budapest, Hungary), was used to obtain the latitude/longitude of the survey locations at harvest. To correct these position data by post-processing [35], RTKLIB ver. 2.4.2 [36], an open-source GNSS position correction program, was used. The observational data (RINEX ver. 3.02) of the electronic reference point (Tsu, station no. 940064) were downloaded from the Electronic Reference Point Data Providing Service of the Geospatial Information Authority (GSI) [37] and loaded into the program.

#### 2.1.3. Soil Sampling and Chemical Properties

Soil samples were collected from the plowed layer at 20 locations in Field A and at 15 locations in Field B on 26–27 March 2019, as shown in Figure 1. Samples were collected when it had not rained for at least three days prior to sampling. The soil samples were air-dried and sieved through 2 mm mesh. pH and electrical conductivity (EC) were measured using a continuous measurement method [38]. Soil water to determine SAN content was extracted according to the procedure of the National Agriculture and Food Research Organization (NARO) [39], and total organic carbon (TOC) was measured in the extracted water using a TOC Analyzer TOC-V CPH (Shimadzu, Kyoto, Japan) and converted to SAN using a calibration curve proposed by NARO. TC and total nitrogen (TN) were measured with the CHNS Elemental Analyzer Vario El cube (Elementar, Langenselbold, Germany), using oven-dried soil samples sieved through 1 mm mesh. Similar to the rice growth survey, we obtained the latitude/longitude of the soil sampling locations by RTK-GNSS and corrected them using a post-processing method.

### 2.2. Image Processing and Analysis

#### 2.2.1. UAS Platform

RGB and multispectral images were taken within two days of the soil sampling and the rice growth survey. The RGB images were taken at an altitude of 30 m using a Phantom 4 Pro (DJI Technology Co. Ltd., Shenzhen, China). The multispectral images were taken at an altitude of 60 m using a Sequoia (Parrot, Paris, France), which captured green (530–570 nm), red (640–680 nm), Red edge (730–740 nm), and NIR (780–810 nm) bands, and was mounted on a Solo (3D Robotics, Berkeley, CA, USA). The shots were taken between 9:30 A.M. and 2:00 P.M. on days when wind speeds were <4 m/s under conditions of low to light cloud cover. In particular, the soil surface images were taken when it had not rained for at least three days to reduce the effects of soil water content. The shooting conditions for both aircraft were 80% front overlap and 75% side overlap with reference to Hama et al. [40] and Micasense [41]. When shooting with the Sequoia, calibration reflectance panels (CRP) were taken before and after the flight and used to convert reflectance. Ground control points (GCPs) were set up at eight locations around the shooting area, and the latitude, longitude, and elevation at each location were obtained using the RTK-GNSS receiver, REACH.

**Figure 2:**
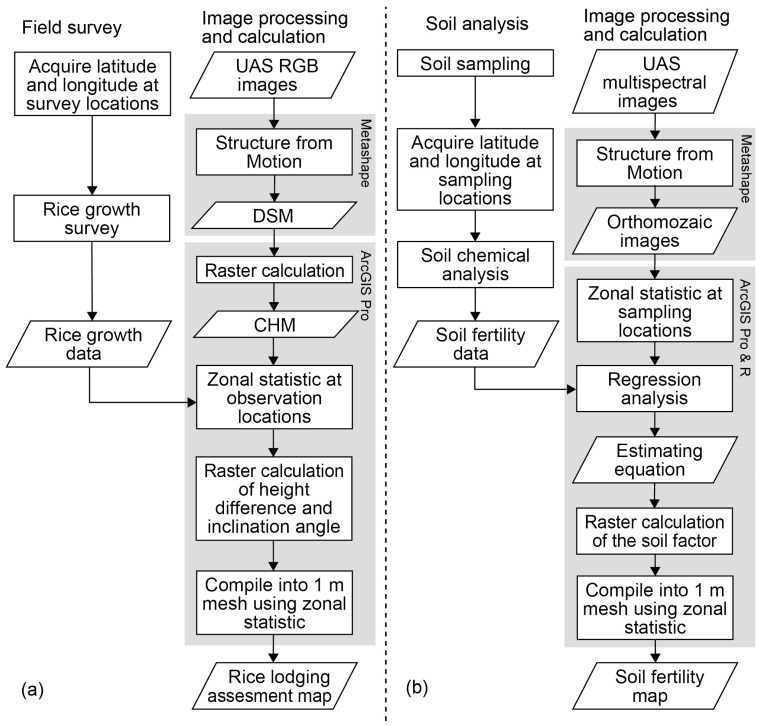
Flowchart of (**a**) rice lodging assessment and (**b**) soil fertility estimation. UAS: unmanned aircraft system; DSM: digital surface model; CHM: canopy height model.

Figure 2 presents a flowchart of the image analysis. The UAS-acquired images were converted into DSM and orthomosaic images using the structure from motion (SfM) processing software Metashape Professional ver. 1.5.5 (Agisoft LLC, St. Petersburg, Russia). The position of the image was corrected using GCPs to improve the accuracy of the positioning. The ground sample distance (GSD) of the RGB orthomosaic image was 1.2 cm/pix, and its multispectral band images were 5.1 cm/pix. The GSD of DSM from the RGB image was 5.0 cm/pix.

#### 2.2.2. Assessment of Rice Growth and Lodging

Figure 2a presents a flowchart of the rice lodging assessment with the UAS images. The DSM generated by Metashape’s SfM was imported into the geographic information system (GIS) software ArcGIS Pro ver. 2.4 (Esri, Redlands, CA, USA). The canopy height model (CHM) was obtained by subtracting the digital terrain model (DTM) as ground elevation using the raster calculation function:

CHM = DSM − DTM(1)

To compare the CHM with the measured plant height, we created buffer polygons with a radius of 0.15 m at all survey locations and calculated the mean values of 60 pixels in each buffer circle using zone statistics. We selected two CHMs for maximum and harvest with reference to the measured plant height data. The difference in plant height between these periods was determined as *δ* CHM. The inclination angle of the rice plants at harvest was determined by the arcsine of the raster calculation function.

*δ* CHM = *r* − *y* (2)

Inclination angle (θ) = arcsine (*y*/*r*) (3)

where *r* is the maximum CHM and *y* is the harvest CHM. We assumed that plant length at harvest was the same as the maximum. The results were compiled into a 1 m mesh to create the rice lodging map.

#### 2.2.3. Estimate of Soil Fertility

Figure 2b presents a flowchart of the soil fertility assessment with the UAS images. The digital number (DN) values of each band image were converted into reflectance using CRP, then orthomosaic images were created through SfM processing. These output images were imported into ArcGIS Pro, and raster calculations were performed. Buffer circles with radii of 0.15, 0.25 and 0.5 m were generated at the soil sampling points to obtain reflectance-converted DNs of the pixel mean values using zone statistics. The explained variables were the results of the soil analysis, and the explanatory variables were the DN of each band, the spectral indices and the normalized difference index (NDI) [42,43].

NDI(*R_i_*, *R_j_*) = (*R_i_* − *R_j_*)/(*R_i_* + *R_j_*) (4)

where *R_i_* is a DN of the *i*-th band’s mean pixel value and *R_j_* is the same for the *j*-th band.

Regression analysis was repeated using the statistical software R ver. 4.0.2 (The R Foundation for Statistical Computing, Vienna, Austria) and the estimated SAN value was rasterized in ArcGIS Pro. The results were compiled into a 1 m mesh to create a soil fertility map using zone statistics.

## 3. Results

### 3.1. Growth of Rice Plants

**Figure 3:**
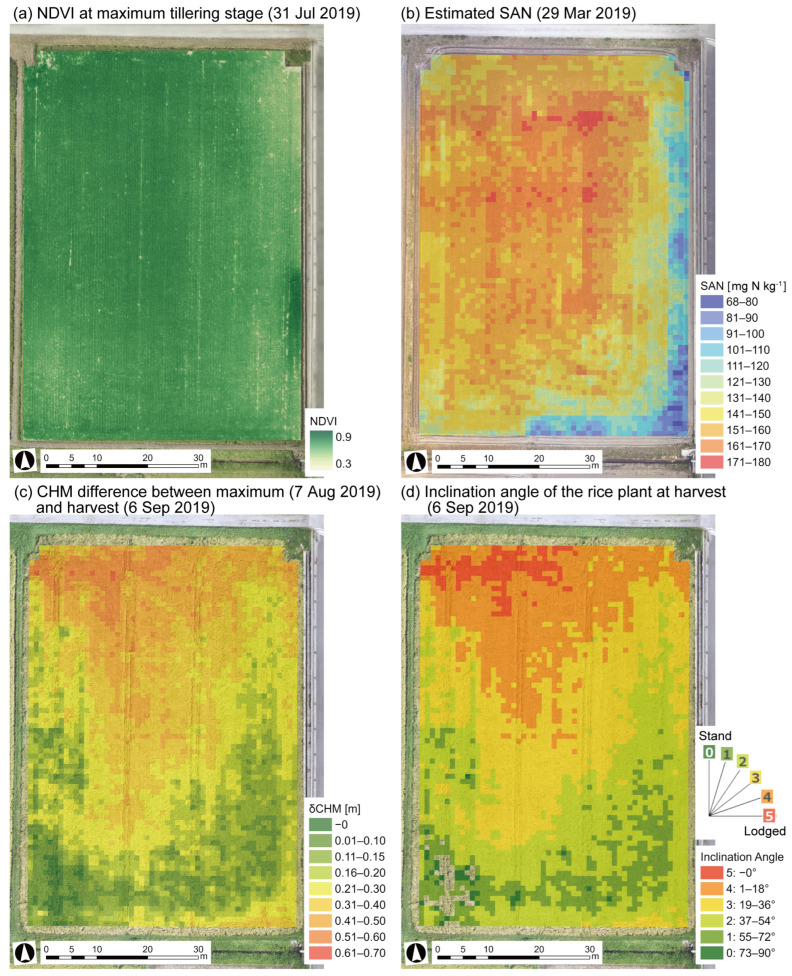
Comparison between (**a**) rice growth status assessed by the NDVI, (**b**) estimated SAN distribution, and (**c**,**d**) lodging severity. NDVI: normalized difference vegetation index; SAN: available nitrogen in soil; CHM: canopy height model.

The NDVI analysis of rice growth in Field A from the UAS images taken on 31 July indicated higher plant activity in the north and less activity in the southeast, as shown in Figure 3a.

**Figure 4:**
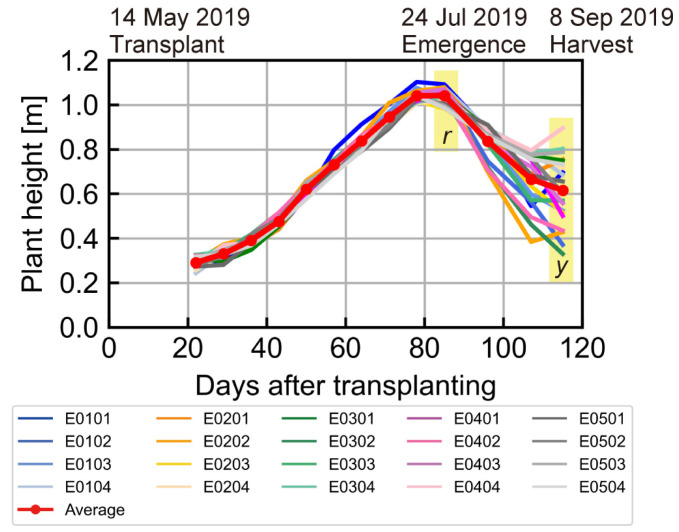
Variation in rice plant height during the research period (June–September 2019). *r*: maximum plant height; *y*: harvest plant height.

Figure 4 shows the rice plant height in Field A. The maximum height of 1.04 m (mean of 20 points) was reached on 7 August, 80 days after transplant. Lodging began to occur on the northwest side of the field 100 days after transplant. There were several rain events in August, the ripening period of the rice. This could have caused lodging by making the ear became heavier. On 6 September (harvest), plant height ranged from 0.33 to 0.90 m with large variation in the stand and lodged locations.

**Figure 5:**
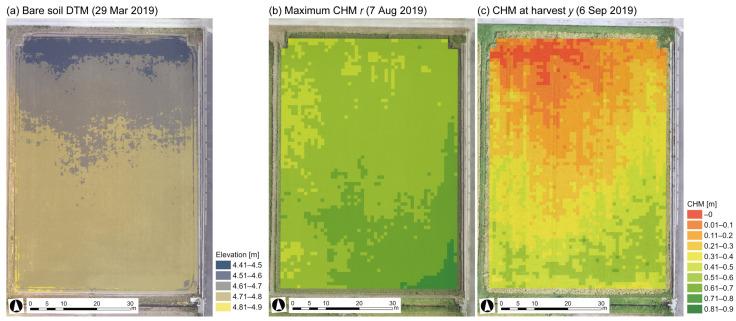
(**a**) Bare soil DTM, (**b**,**c**) calculated maximum plant height and harvest plant height using DSM. DTM: digital terrain model.

Maximum CHM *r* and harvest CHM *y* were obtained by Equation (1) with DSM on 7 August and 6 September, respectively. DTM of the bare soil ground elevation was taken on 29 March 2019, shown in Figure 5a. The elevation range of this field was approximately 4.6–4.8 m and micro-topographically flat, as the slope was calculated to be 0.15°. The calculated CHM *r* and *y* are shown in Figure 5b,c. The range of CHM *r* was about 0.5–0.7 m; on the other hand, CHM *y* varied from 0 to 0.5 m. This showed that CHM was lower and had a wider range of values at the harvest time.

**Figure 6:**
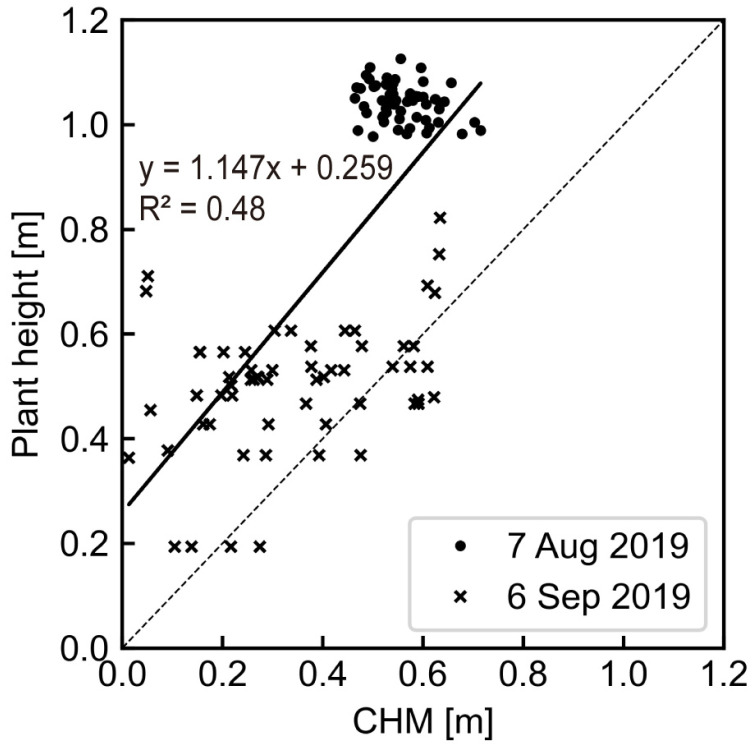
Correlation between measured plant height and the CHM.

Figure 6 compares the measured plant height to the CHM at each survey location. Overall, CHM tended to underestimate plant height by approximately −0.26 m. Wilke et al. [44] reported that UAS CHM was underestimated by 0.01–0.29 m in barley test plots. These deviations were larger in the low-sowing-density plots due to detection of the soil surface. Tanaka and Kondoh [20] added 0.2 m to the CHM values as a correction coefficient to transform plant length when they assessed a potential risk map of rice lodging using plant length. In this study, we applied the calculated CHM to Equations (2) and (3) without correction because the same deviation value was added to *y* and *r* in Equation (2). The difference in the inclination angle was estimated to be about −5° in Equation (3), and it had less impact on the assessment, as rice lodging was evaluated at six levels in 18° increments (Figure 3d); that is, zero for no lodging to five for completely lodged [10].

### 3.2. Assessment of Lodging Severity

Substituting *r* and *y* into Equations (2) and (3), we obtained the difference in plant height and the inclination angle at harvest (Figure 3c,d). The difference in CHM was large on the north side (0.4–0.8 m) and small on the south side (0–0.2 m) (Figure 3c). The north side had a lodged level of 4–5 at less than 18° and the south side had a lodged level of 0–1 at 55–90° (Figure 3d). This heavy lodging on the northwest side and no lodging on the south side was consistent with the visual field observations.

### 3.3. Soil Chemical Analysis

Soil analysis was performed on 20 samples from Field A and 15 samples from Field B, for a total of 35 soil samples collected in March. Table 1 lists the mean, standard deviation (SD) and the coefficient of variation (CV) of the soil physicochemical properties. Both fields had gray lowland soils ranging from silty clay to heavy clay. Field B had a pH of 6.2, while Field A had a pH of 5.7. The TN values were similar in the two fields: Field A had a TC of 2.0%, which was slightly lower than Field B (2.1%), resulting in C/N ratios of 11.5 and 12.9 in Fields A and B, respectively. The SAN content at the 35 different locations varied from 78 to 200 mg N kg^−1^, but the values were generally in the optimum range of 80–200 mg N kg^−1^ [39]. The mean SAN in Field A was 156 mg N kg^−1^, higher than that in Field B at 109 mg N kg^−1^. TC, TN, C/N and SAN varied randomly in the two fields. This result indicated that the chemical characteristics of the soil were not uniform and that the organically cultivated Field B had more varied characteristics.

### 3.4. Estimate of the SAN Distribution

Regression analysis was performed with the reflectance-converted DN of the UAS multispectral image pixel values and the soil chemical properties. Table 2 shows the results of single regression analysis with TC, TN and SAN as the explained variables and the DNs, spectral indices and NDI within a 0.5 m radius buffer circle as the explanatory variables. The cases of 0.15 m and 0.25 m buffer circles were also analyzed, but both resulted in low correlation coefficients, so the results of the 0.5 m case are presented here. In the single regression analysis, TN had a low correlation coefficient of <0.3 with all of the explanatory variables. The correlation coefficient between TC and red–NIR was 0.57.

**Figure 7:**
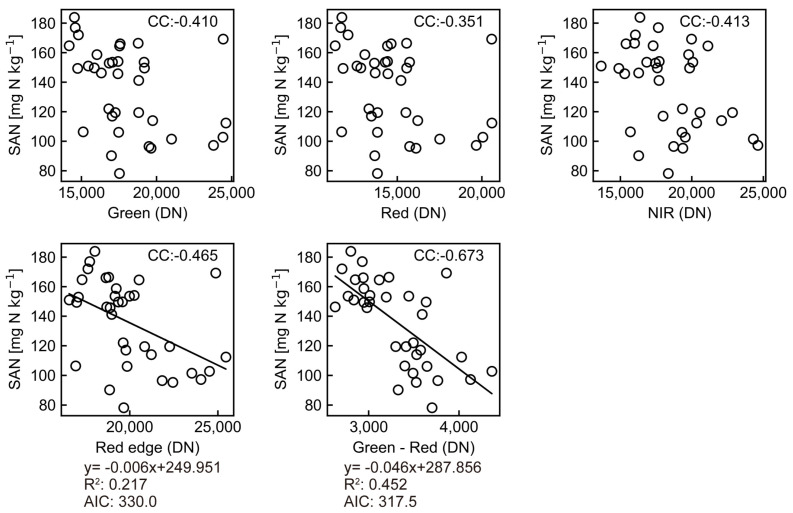
Relationship between SAN and DN of selected explanatory variables with the SAN estimating equation. DN: digital number; CC: correlation coefficient; R^2^: root mean square; AIC: Akaike’s Information Criterion.

As shown in Figure 7, the correlation coefficient between Red edge and SAN was −0.46 in the single band, and the green–red spectral index had the highest correlation coefficient of −0.67. Therefore, we attempted a regression analysis of SAN using green–red as the explanatory variable and obtained the best estimate equation with a coefficient of determination of 0.45 (Figure 7).

SAN = 287.86 − 0.046 × (green − red)(5)

The estimated SAN was rasterized, and these means were inputted to a 1 m mesh, as shown in Figure 3b. The SAN content in Field A was generally adequate (80–200 mg N kg^−1^), but the distribution was higher on the north and center and lower on the southeast side.

## 4. Discussion

### 4.1. Correlation between Rice Lodging and SAN

**Figure 8:**
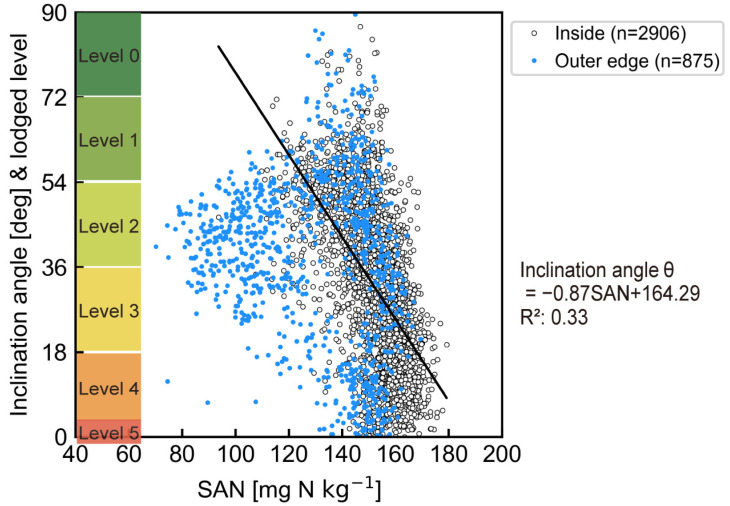
Correlation between SAN and inclination angle of the rice plants at harvest (*n* = 3781). The correlation equation was estimated from inside mesh data (*n* = 2906).

We compared the rice lodging maps with the estimated SAN map for Field A (Figure 3). The degree of lodging was greater from the north to the center of the field and this area roughly coincided with the higher SAN area. The correlation between the rice inclination angle and estimated SAN was verified (Figure 8) by extracting 3781 1 m mesh values for each. The smaller inclination angle of the rice plant indicates a more severe lodged state. A negative correlation was observed between SAN and inclination angle θ for the meshes inside (*n* = 2906), including about 77% of all meshes. This suggests that a higher SAN can cause severe lodging. The optimum SAN level for rice cultivation was 80–200 mg N kg^−1^, but rice lodging occurred at ≥140 mg N. A total of 70 kg N ha^−1^ of one-shot basal fertilizer was applied to Field A, and it corresponded to 70 mg N kg^−1^, assuming that the soil bulk density was 1.0 Mg m^−3^ in the 0–0.1 m tillage layer. It was estimated that the amount of absorbable nitrogen (SAN + fertilizer nitrogen) in the severely lodged meshes was 210–250 mg N kg^−1^. Controlling the amount of absorbable nitrogen to <200 mg N kg^−1^ is desirable in Field A when applying a one-shot basal fertilizer to Koshihikari. The amount of nitrogen eluted differs by soil type and is greatly affected by temperature. In addition, other factors such as soil physicochemical properties, water management, rice variety and cropping system, and their interactions would affect rice growth and lodging in a complex way. Therefore, case studies are needed to obtain feasible guideline values under a variety of conditions.

The blue mark in Figure 8 was the mesh included in the 4 m outer edge of the field (*n* = 875). Many of these data differed in trend from inside meshes data and were excluded when analyzing the correlation between rice inclination angle and SAN. The rice plants in these meshes were observed to be lodged regardless of the SAN content. The degree of lodging was affected by overlapping applications of basal fertilizer because the transplanter moved back and forth in this area.

### 4.2. Factors Influencing the Accuracy of the SAN Estimating Equation

**Figure 9:**
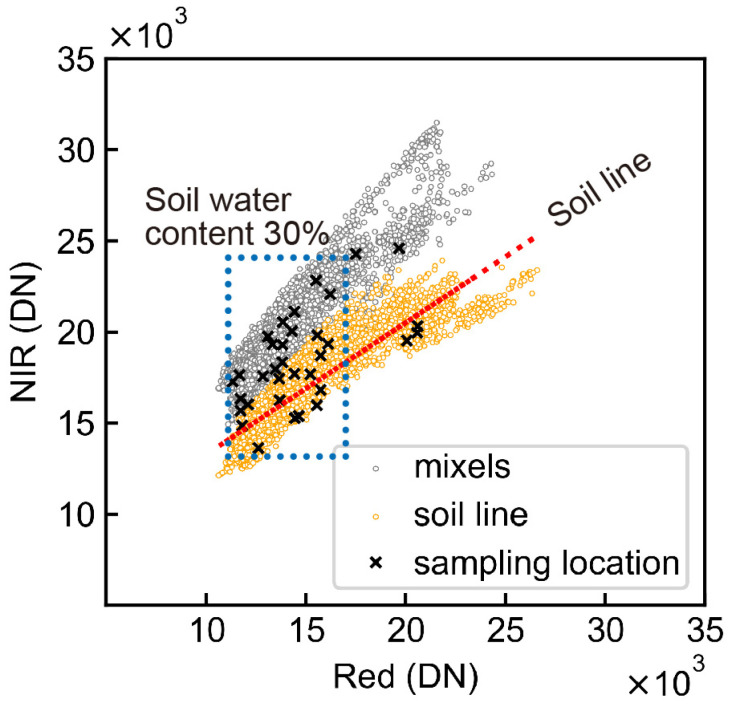
Subplot of red and NIR bands with soil line.

The reflectance-converted DN of the NIR and red bands of bare soil were plotted to verify the accuracy of the SAN estimating equation in Figure 9. The bare soil reflectance of these bands bears a linear relationship called the soil line. The plot shows higher NIR and lower red reflectance as vegetation density increases [32]. The plots in Figure 9 were divided into two groups, so we attempted to classify them by logistic regression [45].

(6) $$p=\frac{1}{1+e^{-(\alpha +\sum \beta_{i}X_{i})}}$$

where *p* is the probability of the soil line pixel varying from 0 to 1, *α* is the intercept of the model, *β_i_* (*i* = 1–4) is the estimated slope coefficient of each variable, and *X_i_* (*i* = 1: green, 2: red, 3: Red edge, 4: NIR) represents the band’s DN as the independent variable.

The orange group was classified as the “soil line,” which did not contain vegetation in the pixels. The gray group showed higher NIR values, which seemed to contain vegetation, such as rice plant residues. These residues caused mixed pixels (mixels) of soil and vegetation on the UAS images and made it difficult to separate the soil pixels. Additionally, the x mark indicates the DN of the soil sampling locations, and its distribution was clustered at lower values. Wet soil has lower NIR and red reflectance, whereas dried soil has higher values [32]. The mean soil water content in the samples was 31.1% (Table 1), which suppressed soil reflectance. These bare soil conditions resulted in low accuracy of the SAN estimating equation. To overcome these issues, we suggest the following measures:

1. Conduct multiple tillage during fallow periods to decompose rice residues.
2. Collect soil sample and take UAS images when the soil is sufficiently dry.
3. Take UAS images when the soil surface is uniform after irrigating the paddy fields [46].
4. Analyze several soil surface images (described above) by machine learning [28].

Moreover, the SAN estimation would be affected by several environmental factors such as soil type, soil physicochemical properties, cropping system, water management and micro-topography. Therefore, further verification of the SAN estimating equation with data obtained in different paddy fields is needed to improve its accuracy.

## 5. Conclusions

The ground elevation of the paddy field where Koshihikari was grown and the time series of the rice plant height were calculated by analyzing DSM with UAS images to determine lodging severity. A SAN-estimating equation was developed with UAS multispectral images of bare soils and represented the spatial distribution of SAN in the paddy field. There was a negative correlation between the inclination angle, which indicates the lodging severity, and SAN. The possibility of lodging was suggested in cases of SAN ≥ 140 mg N kg^−1^, although it was within the optimum range (80–200 mg N kg^−1^). Therefore, it is desirable to control fertilizer to less than 200 mg N kg^−1^ for absorbable nitrogen (SAN + fertilizer nitrogen) in the field when growing Koshihikari using the one-shot basal fertilizer strategy.

Furthermore, the result of the spatial distribution of rice lodging severity suggested that rice plants are particularly prone to lodge at the outer edge of the field, where the transplanter moves back and forth and turns such that overlapping application of fertilizers occurs. When applying basal fertilizer simultaneously with a transplanter, it is recommended to stop fertilization on the return at the outer edge regardless of the SAN content to reduce the amount of fertilizer applied.

In this study, we used a simple linear regression and obtained a SAN estimating equation with the spectral index acquired from UAS multispectral images. The possible measures to improve the accuracy of SAN estimate are acquiring UAS images that minimize the effect of crop residue mixels and soil water contents as well as conducting a machine learning analysis. Further studies on topics such as decomposing crop residues by multiple tillage and taking UAS images under different soil surface conditions are needed. Moreover, rice lodging severity and SAN estimation differ in soil type, soil physicochemical properties, water management, rice variety, cropping system and topography. It is necessary to collect data from various paddy fields and verify this analysis scheme in future study. We aim to generalize this method so that agricultural extension workers can use it practically in the field.

## Figures and Tables

**Table 1:** Soil chemical properties of the study fields.

Soil Indicators		Field A	Field B	All
Depth of Plow Layer [mm]	Mean	104.3	140	119.6
	SD	11.27	19.55	23.46
	CV	10.8	14.0	19.6
Water Content [%]	Mean	31.6	30.5	31.1
	SD	1.27	2.45	1.92
	CV	4.0	8.0	6.2
pH	Mean	5.67	6.21	5.90
	SD	0.187	0.157	0.320
	CV	3.3	2.5	5.4
EC [dS m^−1^]	Mean	0.07	0.10	0.08
	SD	0.025	0.025	0.028
	CV	33.8	24.9	32.8
SAN [mg N kg^−1^]	Mean	156.0	108.5	135.6
	SD	14.10	20.54	29.21
	CV	9.0	18.9	21.5
Total Nitrogen [%]	Mean	0.17	0.16	0.17
	SD	0.012	0.015	0.011
	CV	7.3	9.2	8.3
Total Carbon [%]	Mean	1.99	2.12	2.04
	SD	0.158	0.255	0.212
	CV	8.0	12.0	10.4
C/N	Mean	11.5	12.9	12.1
	SD	0.66	0.80	0.97
	CV	5.7	6.3	8.0

SD: standard deviation; CV: coefficient of variation %; EC: electrical conductivity; SAN: available nitrogen in soil.

**Table 2:** Correlation coefficients of single regression analysis between the soil chemical properties and pixel values of multispectral images.

Explanatory Variables		Explained Variables		
		TN	TC	SAN
Single Band	Gr	−0.094	0.179	−0.410
	Red	−0.055	0.191	−0.351
	RE	−0.093	0.216	−0.465
	NIR	−0.260	−0.184	−0.413
Spectral Index	Gr − Red	−0.300	0.071	−0.673
	Gr − RE	−0.038	−0.004	−0.050
	Gr − NIR	0.167	0.403	−0.039
	Red − RE	0.077	−0.033	0.212
	Red − NIR	0.232	0.413	0.083
	RE − NIR	0.260	0.566	−0.020
	Gr + Red	−0.076	0.185	−0.383
	Gr + RE	−0.095	0.200	−0.446
	Gr + NIR	−0.193	0.007	−0.460
	Red + RE	−0.075	0.208	−0.417
	Red + NIR	−0.179	0.000	−0.430
	RE + NIR	−0.192	0.008	−0.468
	Gr/Red	−0.261	−0.156	−0.265
	Gr/RE	−0.055	0.012	−0.134
	Gr/NIR	0.154	0.379	−0.050
	Red/RE	0.019	0.056	−0.051
	Red/NIR	0.183	0.383	−0.006
	RE/NIR	0.256	0.533	0.022
NDI	Gr, Red	−0.261	−0.159	−0.263
	Gr, RE	−0.063	0.015	−0.148
	Gr, NIR	0.147	0.376	−0.065
	Red, RE	0.012	0.054	−0.062
	Red, NIR	0.177	0.377	−0.020
	RE, NIR	0.264	0.535	0.023

Gr, Red, RE, NIR: reflectance converted digital number (DN) in the green, red, Red edge and NIR bands, respectively. NDI: normalized difference index.

## Data Availability

The data presented in this study are available on request from the corresponding author.
